# Real-world effectiveness and safety of 1L polyethylene glycol and ascorbic acid for bowel preparation in patients aged 80 years or older

**DOI:** 10.1055/a-2525-9938

**Published:** 2025-02-26

**Authors:** Salvador Machlab, Vicente Lorenzo-Zúñiga, Miguel Angel Pantaleon, Fernando Sábado, Cátia Arieira, Elena Pérez Arellano, José Cotter, David Carral, Carmen Turbí Disla, Ricardo Gorjão, Jose Miguel Esteban, Sarbelio Rodriguez

**Affiliations:** 1 Gastroenterology, Parc Taulí Hospital Universitari, Institut d’Investigació i Innovació Parc Taulí I3PT, Sabadell, Spain; 2 Gastroenterology, Hospital HM Sant Jordi, Barcelona, Spain; 3 16548 Gastroenterology, Hospital del Mar, Barcelona, Spain; 4 Gastroenterology, Consorcio Hospitalario Provincial de Castelló, Castellón, Spain; 5 Gastroenterology, Hospital da Senhora da Oliveira, Guimarães, Portugal; 6 207202 Gastroenterology, Hospital Universitario La Zarzuela, Madrid, Spain; 7 School of Medicine, Universidade do Minho, Life and Health Sciences Research Institute (ICVS), Braga/Guimarães, Portugal; 8 Gastroenterology, ICVS/3B’s – PT Government Associate Laboratory, Braga/Guimarães, Portugal; 9 Gastroenterology, Hospital San Rafael, A Coruña, Spain; 10 Medical Affairs, Norgine, Harefield, United Kingdom of Great Britain and Northern Ireland; 11 Gastroenterology, Hospital CUF Descobertas, Lisboa, Portugal; 12 Gastroenterology, Hospital Clínico Universitario San Carlos, Madrid, Spain; 13 Gastroenterology, Hospital Ruber Juan Bravo, Madrid, Spain

**Keywords:** Quality and logistical aspects, Preparation, Endoscopy Small Bowel, Performance and complications

## Abstract

**Background and study aims:**

Clinical trials and real-world studies show a 1L polyethene glycol and ascorbic acid solution (1L PEG-ASC) to be an effective and safe bowel preparation for colonoscopy in the general population. Here, the effectiveness and safety of 1L PEG-ASC were evaluated in patients aged 80 years or older in a real-world setting.

**Patients and methods:**

A post-hoc analysis of an observational, multicenter, retrospective study assessed the effectiveness and safety of 1L PEG-ASC on outpatients aged ≥ 80 years old undergoing colonoscopy at eight centers in Spain and Portugal. Cleansing quality was assessed using the Boston Bowel Preparation Scale, with overall scores ≥ 6 and all segmental scores ≥ 2 considered adequate colon cleansing, and overall scores ≥ 8 or 3 in the right colon considered high-quality cleansing. Cecal intubation rate, withdrawal time, polyp and adenoma detection rates (ADR), and adverse events (AEs) were also monitored.

**Results:**

Data were analyzed from 423 patients aged ≥ 80 years; mean age 83.5 years (±3.2) and 49.2% males. The adequate colon cleansing success rate was 88.9%, with high-quality cleansing of the overall and right colon achieved in 54.1% and 46.1% of patients, respectively. Colonoscopy was complete in 94.1% of cases and the ADR was 51.3%. At least one AE was experienced by 4.5% of participants, the most frequent being mild dehydration (2.8%) and nausea (1.2%).

**Conclusions:**

This post-hoc analysis confirms 1L PEG-ASC to be an effective and safe bowel cleansing preparation for patients aged 80 years or older in a real-world setting.

## Introduction

Colonoscopy remains the gold standard to screen and monitor colorectal cancer (CRC) 1 , the incidence of which increases by 80% to 100% in each incremental 5-year age group up to 50 years of age, and by about 30% from 55 to 59 years of age and above 2 . Given the rapid aging of the worldwide population, the number of colonoscopies performed on the elderly is on the rise, making it increasingly important to ensure successful visualization of the colonic mucosa to ensure high-quality colonoscopies are achieved in this population as often as possible. However, the preparation for colonoscopy examinations can be technically challenging in the elderly 3 and frail patients in particular, with success rates ranging from 48% to 94% 4 . Although colonoscopies in patients aged ≥ 80 years old have proven to be safe procedures with a generally high diagnostic yield 3 4 5 6 7 8 9 10 , they are technically challenging examinations, not least due to difficulties in achieving adequate bowel preparation in this population. Indeed, inadequate bowel cleansing has been reported in 12% to 26% of patients in this age group 4 8 10 11 12 13 , and poor bowel preparation is considered to be one of the main factors responsible for the poor completion rates in elderly patients 3 4 8 10 11 . Reasons for a higher likelihood of poor bowel preparation in elderly patients remain unclear. However, it has been speculated that they may have a lower tolerance than younger people to ingesting large volumes of the preparative agents or that it may be due to reduced gastrointestinal motility, greater difficulties in understanding the preparation instructions or more comorbidities 14 . Other reasons may include dietary factors, slower colon transit time, higher incidence of constipation, dehydration, or greater risk of clinically significant electrolyte disturbances 12 .

Colonoscopy quality is dependent on adequate bowel cleansing, which can affect diagnostic accuracy and the adenoma detection rate (ADR) 15 . Indeed, inadequate bowel preparation often results in poor visualization of the mucosa, resulting in reduced sensitivity of colonoscopies, missed lesions, longer procedure times, and higher risk of adverse events (AEs), ultimately enhancing likelihood of having to repeat the examinations with an increase in associated healthcare costs 16 .

Bowel cleansing with polyethylene glycol (PEG) solutions is considered safe in the general geriatric population 17 . An ultra-low-volume bowel preparation solution containing PEG and ascorbic acid (1L PEG-ASC, PLENVU: Norgine Harefield, UK) was developed to improve patient satisfaction during colonoscopy, in part by reducing the total volume of liquids that must be ingested for preparation. In light of the evidence from three phase 3 randomized controlled trials 18 19 20 , this 1L PEG-ASC solution was introduced in Portugal in 2017 and Spain in 2018 to be used for bowel cleansing in adults before any procedure requiring such preparation. Small studies in real-world settings have also confirmed the effectiveness and safety of both low- and high-volume PEG-based preparations and highlighted the improved outcome in both overall and right colon cleansing with use of 1L PEG-ASC 21 22 . Recently, a large multicenter study in Spain and Portugal (13,169 patients) also confirmed the effectiveness and safety of 1L PEG-ASC in the general population in a real-world setting 23 . Because data specific to patients aged ≥ 80 years old are lacking, we set out to perform a post-hoc analysis of this large, multicenter study to evaluate the effectiveness and safety of 1L PEG-ASC bowel preparation in routine clinical practice when used on the population of patients aged 80 years or over.

## Patients and methods

### Study design and participants

A post-hoc analysis was performed on data from an observational, retrospective, multicenter study in Portugal and Spain 23 . Included in this analysis were data from eight centers in Spain and Portugal, evaluating outpatients aged ≥ 80 years old who received 1L PEG-ASC for bowel preparation before undergoing a screening, follow-up or diagnostic colonoscopy between June 2019 and September 2021. The 1L PEG-ASC preparation was taken as recommended in the summary of product characteristics, either following an overnight split-dose (pm/am, i.e., with the first dose taken in the evening on the day before and the second dose in the morning of the day of the test) or a same-day regimen (with both doses taken in the morning on the day of colonoscopy, and with the second dose taken at least 2 hours after the start of the first dose). All patients were instructed to follow a fiber-free diet for at least 24 hours before the preparation and written instructions about how to consume the bowel preparation were provided by each hospital. Exclusion criteria were a history of CRC or colectomy or the impossibility of obtaining the required “mandatory data”: sex; age; indication for colonoscopy; dosing regimen; complete colonoscopy; Boston Bowel Preparation Scale (BBPS) for the right, transverse, or left colon; and number of polyps in each segment.

The Ethical Review Committee approved the study at the Hospital Clínico San Carlos and registered in an international clinical trials registry (ClinicalTrials.gov NCT05174845). The data were collected from anonymized medical records.

### Outcome assessment

The three main endpoints were adequate and high-quality (HQ) overall colon cleansing and HQ right colon cleansing, which were assessed through BBPS score. Adequate bowel cleansing was defined as a BBPS score ≥ 6 with a BBPS score ≥ 2 in each segment, while HQ cleansing was defined as a BBPS score ≥ 8 for the overall colon and a BBPS score of 3 for the right colon.

The main exploratory endpoints were polyp detection rates (PDRs), proportion of colonoscopies where at least one polyp was detected; ADR, the proportion of colonoscopies where at least one adenoma was found and confirmed histologically; cecal intubation rate (CIR); cecal withdrawal time; and safety, assessed from the recorded AEs.

### Statistical analysis

A descriptive analysis was performed for variables of interest, calculating means and standard deviations (SD) for quantitative variables. For categorical variables, frequencies and percentages were calculated. Analyses were performed with SAS for Windows (V9.4 or later: SAS Inc., North Carolina, United States). Because this study is descriptive, no sample size calculations were performed.

## Results

### Baseline demographics and clinical characteristics of participants

Data from 423 patients aged ≥ 80 years old were available, with a mean age of 83.5 ± 3.2 years (median 83.0; range 80–95), of whom 49.2% were male ( Table 1 ). Main indications for colonoscopy were diagnosis (56.7%), follow-up (25.5%), screening for CRC (14.4%) or other (3.3%). Where data were available, the most frequent comorbidities were the following: hypertension (85.5%), pelvic or abdominal surgery (40.0%), and diabetes mellitus (39.4%; Table 1 ). A same-day dose regimen was administered to 59.1% of the participants and an overnight split-dose regimen to 40.9%.

**Table:** **Table 1** Demographic and clinical characteristics.

	**Total (N = 423)**
**Sex, n (%)**	
Male	208 (49.2%)
Female	215 (50.8%)
**Mean age, years (SD)**	83.46(3.2)
**Main comorbidities**	
**Hypertension**	118/138 (85.5%)
**Pelvic or abdominal surgery**	38/95 (40.0%)
**Diabetes mellitus**	54/137 (39.4%)
**Mild/moderate renal impairment**	33/137 (24.1%)
**Mild/moderate kidney failure**	33/137 (24.1%)
**Constipation**	24/137 (17.5%)
SD, standard deviation.	

### Bowel cleansing effectiveness

**Fig. 1:**
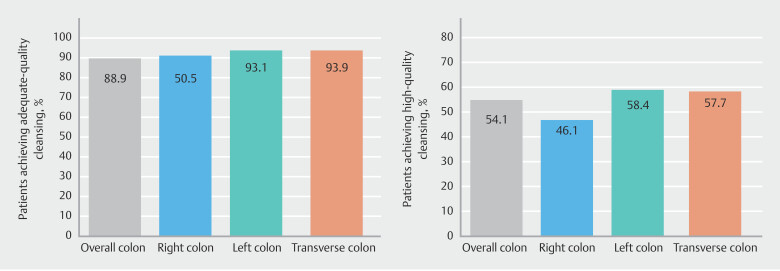
**a** Proportion of participants that achieved adequate cleansing of the overall colon (BBPS ≥ 6 with a BBPS ≥ 2 in each segment) and of individual segments (BBPS ≥ 2). **b** Proportion of patients achieving HQ cleansing of the overall colon (BBPS ≥ 8) and of individual segments (BBPS = 3).

**Fig. 2:**
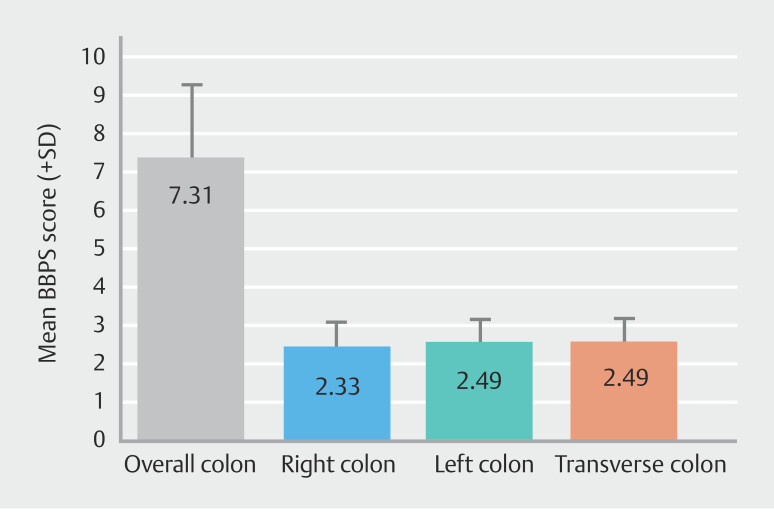
Cleansing scores according to mean Boston Bowel Preparation Scale (BBPS) for overall colon and for individual segments. Error bars reflect standard deviation.

The adequate overall colon cleansing success rate with 1L PEG-ASC was 88.9% ( Fig. 1 **a** ), with HQ cleansing in the total colon achieved in 54.1% of patients ( Fig. 1 **b** ) and HQ cleansing in the right colon achieved in 46.1% of patients ( Fig. 1 **b** ). Mean BBPS was 7.31 (± 1.96) and 2.33 (± 0.76) for the overall and right colon, respectively ( Fig. 2 ). Significantly better cleansing was achieved in the group that underwent a screening colonoscopy, both in terms of adequate cleansing of the overall colon (95.1% vs 85.0% for diagnostic colonoscopy: *P* < 0.05), as well as HQ right colon cleansing (52.5% vs 37.9% for diagnostic colonoscopy: *P* < 0.05). However, there were no differences in overall HQ colon cleansing between screening (60.7%) and diagnostic colonoscopies (48.3%: *P* = 0.08). Also, significantly better adequate overall cleansing was achieved in patients who followed a split-dose regimen (94.8%), as well as better HQ overall cleansing (63.6%) and HQ right colon cleansing (53.2%), relative to those who followed the same-day regimen (adequate overall cleansing, 84.8%; HQ overall cleansing, 47.6%; and HQ right colon cleansing, 41.2%: *P* < 0.05).

### Polyps and adenoma detection

At least one polyp was detected and removed in almost half the patients undergoing a colonoscopy (45.6%), with a PDR in the right colon of 26.7%. Mean number of polyps detected per patient was 1.07 (± 1.81) in the overall colon and 0.43 (± 0.90) in the right colon. The ADR was 51.3% (135/263) in the overall colon and 38.2% (89/233) in the right colon.

### Other colonoscopy outcomes

Colonoscopy was completed in 94.1% of patients. In 1% of patients, non-completion was due to poor preparation, whereas stenosing cancer (1.4%), technical difficulties (1.2%), and other reasons (2.3%) were also indicated as motives for non-completion. In addition, the CIR was 95.0% (227/239), and mean withdrawal time was 8.4 (± 5.3) minutes (95% CI 7.56–9.18, n = 166).

## Safety

Of the patients, 4.5% (n = 19) experienced at least one AE. The most common AEs were mild dehydration (n = 12, 2.8%: resolved with oral rehydration solutions) and nausea (n = 5, 1.2%), followed by vomiting (n = 2, 0.47%) and dizziness (n = 1, 0.24%). No severe AEs were reported by any of the participants.

## Discussion

Life expectancy has increased in Europe 24 due to improvements in lifestyle and medical care, which has led to a significant increase in colonoscopies performed on patients older than age 80 years. In response to this shift, this is the first analysis to address the effectiveness, safety, and completeness of colonoscopies performed following preparation with a 1L PEG+ASC solution in patients aged 80 years or over in real-life clinical practice. In this real-world population of patients aged 80 years old or older, overall adequate bowel cleansing with the 1L PEG–ASC preparation (88.9%) is close to the 90% minimum adequate cleansing standard recommended in the ESGE (European Society of Gastrointestinal Endoscopy) guidelines 25 , and with over half of the patients achieving HQ total colon cleansing. HQ bowel cleansing improves the ADR and it is required for optimal detection of sessile serrated polyps 26 . A post-hoc analysis of three clinical trials demonstrated that when compared with adequate cleansing of the overall and right colon, HQ cleansing is associated with a higher ADR and a higher mean number of adenomas detected in patients, a measure of increasing importance for CRC prevention 27 .

A recent meta-analysis demonstrated that a 1L PEG-ASC solution produced significantly higher cleansing success and HQ right-colon cleansing rates than other bowel preparations with a similar ADR 28 . In our population of patients aged ≥ 80 years old, HQ right colon cleansing was also greater than that seen in clinical trials 18 19 20 and similar to that in one real-world study targeting all ages 21 . This high right-colon HQ cleansing rate is of primary importance for detecting high-risk sessile serrated polyps, lesions that are more common in this colon segment where the rate of missed lesions is greater 29 . Furthermore, development of dysplasia in sessile serrated polyps has been associated with increasing age 30 . PDR is non-inferior to ADR in predicting the risk of interval CRC 31 , with a minimum standard PDR at 40% corresponding to an ADR of 25% 32 . Nevertheless, ADR is the primary clinical indicator of colonoscopy quality, and it is inversely associated with future risk of CRC and death 27 33 , with enhanced detection of colorectal adenomas associated with HQ colon cleansing 27 . In this analysis, the ADR of 51.3% doubles the minimum ESGE standard of 25% 25 , such that overall adequate and HQ cleansing with 1L PEG–ASC appears to translate into a high ADR, coinciding with the higher prevalence of adenomas at older ages 34 . Thus, these results support reliable performance of 1L PEG–ASC to provide successful colonoscopy outcomes in patients aged 80 years or older, owing to effective HQ bowel cleansing.

Although detection and resection of asymptomatic adenomas have little impact on elderly or morbid patients, detection and treatment of small vascular lesions or angiodysplasias may be important 35 . This type of lesion can lead to transfusion dependency and frequent follow-up visits, generating a considerable burden on health resources and a reduction in quality of life and they are most often found in the cecum or right colon, with their incidence increasing with age 36 . In studies on colonoscopy performance and bowel cleansing, the clinical impact is mainly evaluated using ADR as a surrogate variable for interval cancer and PDR as a predictor of ADR 32 . However, no studies have specifically focused on, nor are there specific indicators for, the clinical impact of colonoscopy in elderly and frail patients. Thus, we based our analysis here on detection rates of lesions, which could be considered parallel to, or even more sensitive than, detection of vascular lesions, particularly given the absence of a benchmark indicator such as an angiodysplasia rate.

Colonoscopy completion rate, another quality performance indicator, was high in our study (94.1%), and poor preparation was the reason for non-completion in only a small proportion of cases (1%). This figure is only slightly higher than the 0.8% from the analysis targeting the general population 23 and lower than that reported in another real-life study 22 . A meta-analysis of 20 studies reported poor bowel preparation in 12.1% of patients aged ≥ 80 years old, with a mean colonoscopy completion rate of 84.7% 11 . For patients aged ≥ 85 years, a 69% colonoscopy completion rate was reported, mostly attributed to poor bowel preparation 13 . Moreover, a prospective study comparing octogenarians and non-octogenarians showed a lower colonoscopy completion rate in the former (90% vs 99%), which was again related to poorer quality of colon preparation 4 . For patients aged ≥ 90 years old, colonoscopies were completed in 88.2%, again associated with a higher incidence of inadequate bowel preparation 37 . Thus, our findings represent an improvement over previous studies reporting considerably lower completion rates due to poor preparation in octogenarians and nonagenarians following various bowel preparations. Perhaps the most important determinant of inadequate bowel preparation in elderly patients is not chronological age or the regimen used but rather, the higher incidence of comorbidities that are associated with aging. As such, a patient in their 90s in relatively good health would be more likely to achieve adequate bowel preparation than a patient in their 70s with two comorbid conditions like diabetes and Parkinson’s disease.

Low CIR has been associated with increased risk of interval CRC 38 , and a CIR < 80% with a significantly higher risk of proximal and distal interval CRCs compared with higher completion rates 38 . Here, a high (95.0%) CIR was achieved that exceeded the minimum standard (90%) recommended in the ESGE guidelines for overall indications, and that equals the target recommended in those guidelines. In this study, the mean withdrawal time was 8.4 minutes, the same as that in the general population 23 , and it also surpassed the minimum standard recommended in the ESGE guidelines 25 . However, it should be noted that data on complete cecal intubation and withdrawal time were not recorded for 43.5% and 60.7% of the participants, respectively.

Incidence of AEs was low (4.5%) in our population of patients aged 80 years and over, with mild dehydration the most common AE reported, consistent with the higher vulnerability to dehydration of the elderly than younger patients. This figure is considerably lower than in clinical trials 18 19 20 and one prospective real-life study 22 . This may be due to the solicited reporting of AEs in clinical trials, as opposed to spontaneous reporting in retrospective real-world studies. This phenomenon may lead to minor AEs being perceived as unimportant and unreported. In addition, data collection in prospective real-world studies is monitored and recorded by trained personnel, which might result in more frequent reporting of AEs in prospective studies than in retrospective ones.

A strength of this post-hoc analysis is that it provides a representative overview of cleansing results within a population of patients aged 80 years old and over. It includes participants from six hospitals in Spain and two in Portugal, thereby providing a good representation of patients in this age group, with various comorbidities and in whom colonoscopies were performed following different clinical practices. The retrospective design makes it easier and quicker to analyze real-world data, even though this can also be achieved in prospective studies, and this approach provided evidence of performance of bowel preparations in clinical practice, an important issue when designing strategies for bowel cleansing in clinical settings. However, the retrospective, observational nature of the study may also be considered a limitation because it implies some clinical information may be missing, such as data regarding comorbidities, as indicated above. Nevertheless, in the present study, no data were missing for the outcome measurements collected as primary endpoints in daily clinical practice and our analysis focused mainly on procedure endpoints consistently collected in daily practice. Furthermore, and as indicated above, it is possible that some AEs may not have been recorded in participant clinical records, a failure in analysis of retrospective data based on medical histories. Finally, it should be noted that no direct comparisons were made with the population aged < 80 years old or with other cleansing preparations that might be used in clinical practice, because our analysis focused solely on outcome measures regarding performance of 1L PEG–ASC in patients aged 80 years old or over in daily clinical practice.

## Conclusions

In conclusion, this post-hoc analysis supports the effectiveness of the 1L PEG-ASC preparation to attain adequate overall and HQ colon cleansing, as well as HQ right colon cleansing in patients aged 80 years or over in a real-world setting and with a good safety profile.
