# Correction: Real-world effectiveness and safety of 1L polyethylene glycol and ascorbic acid for bowel preparation in patients aged 80 years or older

**DOI:** 10.1055/a-2822-5767

**Published:** 2026-03-02

**Authors:** Salvador Machlab, Vicente Lorenzo-Zúñiga, Miguel Angel Pantaleon, Fernando Sábado, Cátia Arieira, Elena Pérez Arellano, José Cotter, David Carral, Carmen Turbí Disla, Ricardo Gorjão, Jose Miguel Esteban, Sarbelio Rodriguez

**Affiliations:** 1 Gastroenterology, Parc Taulí Hospital Universitari, Institut d’Investigació i Innovació Parc Taulí I3PT, Sabadell, Spain; 2 Departament de Medicina, Universitat Autònoma de Barcelona, Cerdanyola del Vallès, Spain; 3 Gastroenterology, Hospital HM Sant Jordi, Barcelona, Spain; 4 16548 Gastroenterology, Hospital del Mar, Barcelona, Spain; 5 Gastroenterology, Consorcio Hospitalario Provincial de Castelló, Castellón, Spain; 6 Gastroenterology, Hospital da Senhora da Oliveira, Guimarães, Portugal; 7 207202 Gastroenterology, Hospital Universitario La Zarzuela, Madrid, Spain; 8 School of Medicine, Universidade do Minho, Life and Health Sciences Research Institute (ICVS), Braga/Guimarães, Portugal; 9 Gastroenterology, ICVS/3B’s – PT Government Associate Laboratory, Braga/Guimarães, Portugal; 10 Gastroenterology, Hospital San Rafael, A Coruña, Spain; 11 Medical Affairs, Norgine, Harefield, United Kingdom of Great Britain and Northern Ireland; 12 Gastroenterology, Hospital CUF Descobertas, Lisboa, Portugal; 13 Gastroenterology, Hospital Clínico Universitario San Carlos, Madrid, Spain; 14 Gastroenterology, Hospital Ruber Juan Bravo, Madrid, Spain



In the above-mentioned article the affiliation for Salvador Machlab was corrected. This was corrected in the online version on 3 March 2026.

